# Size variation of infrared vibrational spectra from molecules to hydrogenated diamond nanocrystals: a density functional theory study

**DOI:** 10.3762/bjnano.4.28

**Published:** 2013-04-15

**Authors:** Mudar A Abdulsattar

**Affiliations:** 1 Ministry of Science and Technology, Baghdad, Iraq; Tel. +964-7901335231

**Keywords:** ab initio, diamond, infrared spectroscopy, nanocrystals, vibration

## Abstract

Infrared spectra of hydrogenated diamond nanocrystals of one nanometer length are calculated by ab initio methods. Positions of atoms are optimized via density functional theory at the level of the generalized gradient approximation of Perdew, Burke and Ernzerhof (PBE) using 3-21G basis states. The frequencies in the vibrational spectrum are analyzed against reduced masses, force constants and intensities of vibration. The spectrum can be divided into two regions depending on the properties of the vibrations or the gap separating them. In the first region, results show good matching to several experimentally obtained lines. The 500 cm^−1^ broad-peak acoustical branch region is characterized by pure C–C vibrations. The optical branch is centered at 1185 cm^−1^. Calculations show that several C–C vibrations are mixed with some C–H vibrations in the first region. In the second region the matching also extends to C–H vibration frequencies that include different modes such as symmetric, asymmetric, wagging, scissor, rocking and twisting modes. In order to complete the picture of the size dependence of the vibrational spectra, we analyzed the spectra of ethane and adamantane. The present analysis shows that acoustical and optical branches in diamond nanocrystals approach each other and collapse at 963 cm^−1^ in ethane. Variation of the highest reduced-mass-mode C–C vibrations from 1332 cm^−1^ of bulk diamond to 963 cm^−1^ for ethane (red shift) is shown. The analysis also shows the variation of the radial breathing mode from 0 cm^−1^ of bulk diamond to 963 cm^−1^ for ethane (blue shift). These variations compare well with experiment. Experimentally, the above-mentioned modes appear shifted from their exact positions due to overlap with neighboring modes.

## Introduction

Diamond nanocrystals are a very important material theoretically and experimentally. This importance seems to originate from the extraordinary properties of bulk diamond that include high hardness, inertness and high thermal conductivity. The additional properties added by reduction to the nanoscale make diamonds and related carbon materials a focus for recent investigations [1–9]. One of the first steps of investigating a material is the characterization of its properties. The present work is concerned with the theoretical calculation of vibrational infrared frequency lines of diamond nanocrystals and the variation of these vibrations from molecular to bulk sizes. Several previous calculations from other authors assigned different origins for some of the well observed lines, such as the experimental 500 and 1130–1332 cm^−1^ diamond nanocrystal lines [2–3,6]. In the present work we shall try to explain and calculate some of these lines. In addition, we shall discuss C–H vibrations and their mixing with C–C vibrations. The importance of identifying C–H frequencies will be shortly demonstrated in the subsequent sections. The variation of C–C vibrations with the size of the carbon–hydrogen molecules or nanocrystals is shown by including the investigation of ethane and adamantane molecules.

### Theory

Density functional theory at level of the generalized gradient approximation of Perdew, Burke and Ernzerhof (PBE) is used in the present work to determine stable optimized positions of atoms in the nanocrystal [10]. Double-zeta 3-21G basis functions are chosen to perform the above calculations so that all vibrational analysis is performed with the same level of theory, which is feasible within our computer system in terms of memory and time. The chosen diamond nanocrystal is of 1 nm length. It has the stoichiometry C_64_H_84_. After optimizing geometrical positions, vibrational frequencies are determined by solving coupled perturbed Hartree–Fock equations [11–12]. The frequencies are then analyzed against other vibrational properties such as reduced masses, force constants and infrared vibration intensities. To complete the picture of C–C vibrations with size variation we included the infrared vibrational frequencies of ethane and adamantane molecules using the same level of theory.

## Results

The program Gaussian 03 [13] is used to optimize the geometries and calculate the vibrational spectra of diamond nanocrystal, ethane and adamantane molecules. The calculated frequencies need to be corrected for the systematic frequency error that results from ab initio calculations [10]. The previous estimation of this scale factor for PBE theory by using the 3-21G basis is 0.991 [14]. Note that different authors use different scale factors for the same basis at the same level of calculation [10,14–15]. The present scale factor is one of the nearest scale factors to the unscaled data (very close to 1) and will be used without modification for all spectra.

**Figure 1:**
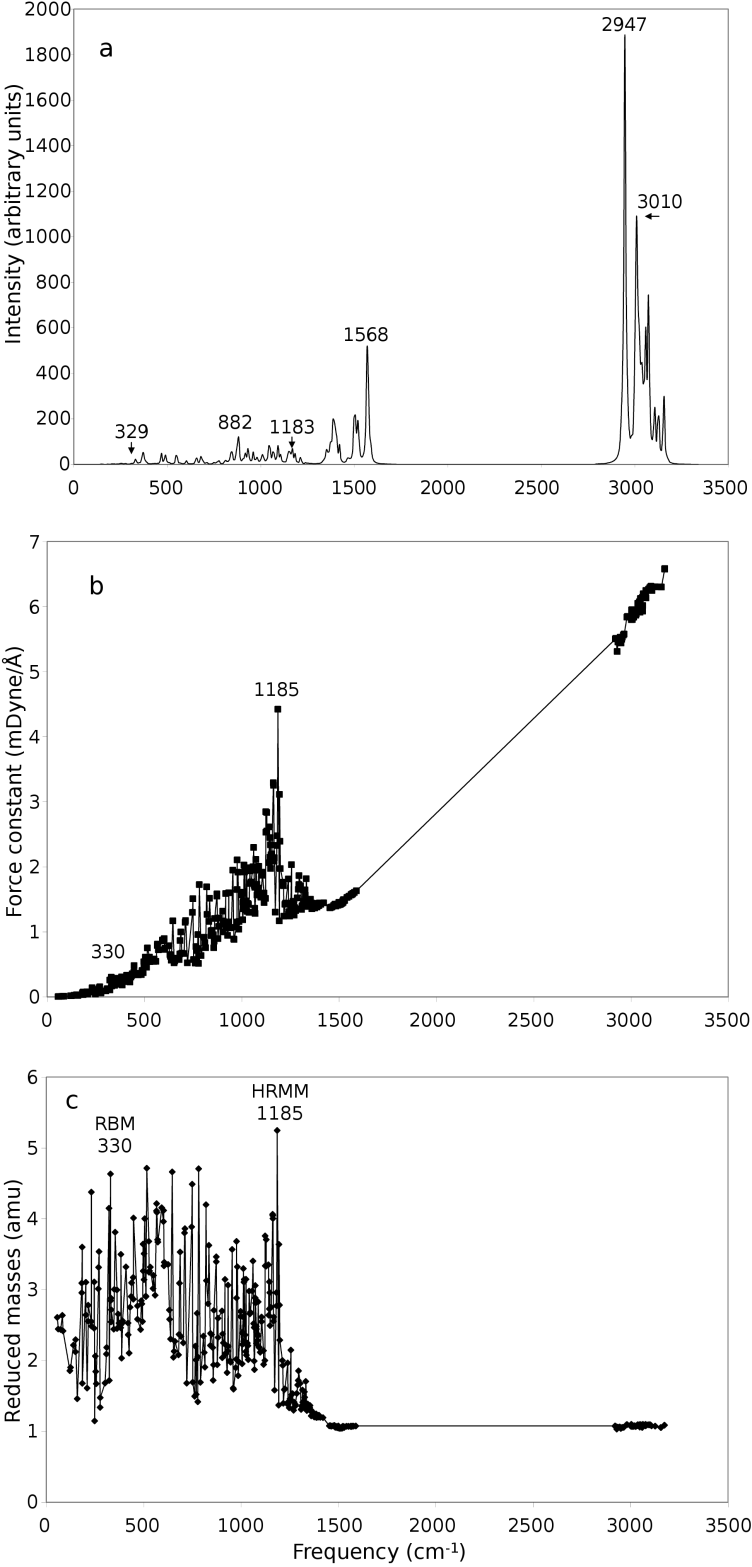
(a) Intensities, (b) force constants and (c) reduced masses of the diamond nanocrystal C_64_H_84_ infrared spectrum as a function of frequency.

Figure 1 shows the vibration frequency of the C_64_H_84_ nanocrystal. This figure and the following two figures include the analysis of vibrational reduced masses, force constants and infrared vibrational intensities against the frequency of vibration.

**Figure 2:**
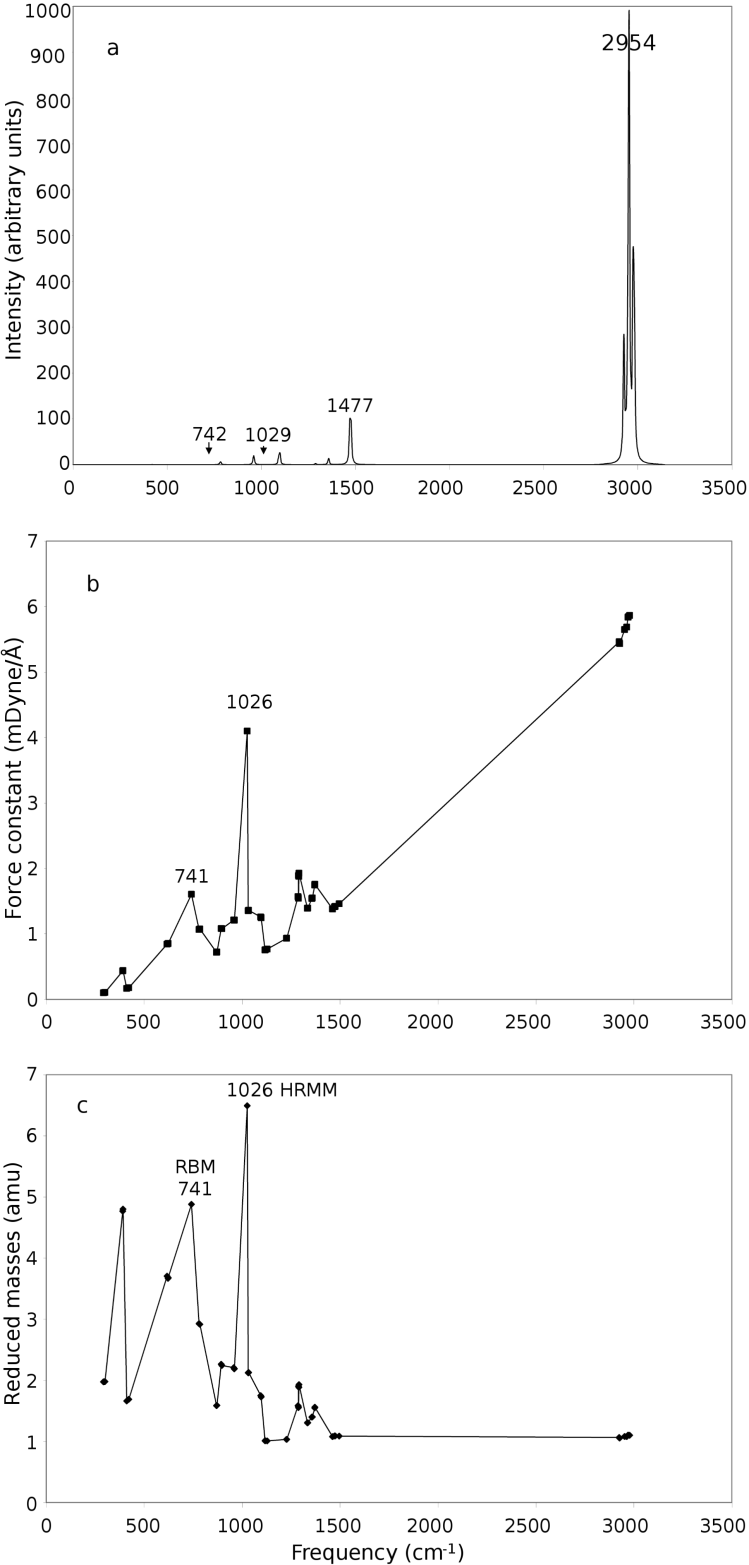
(a) Intensities, (b) force constants and (c) reduced masses of the adamantane infrared spectrum as a function of frequency.

**Figure 3:**
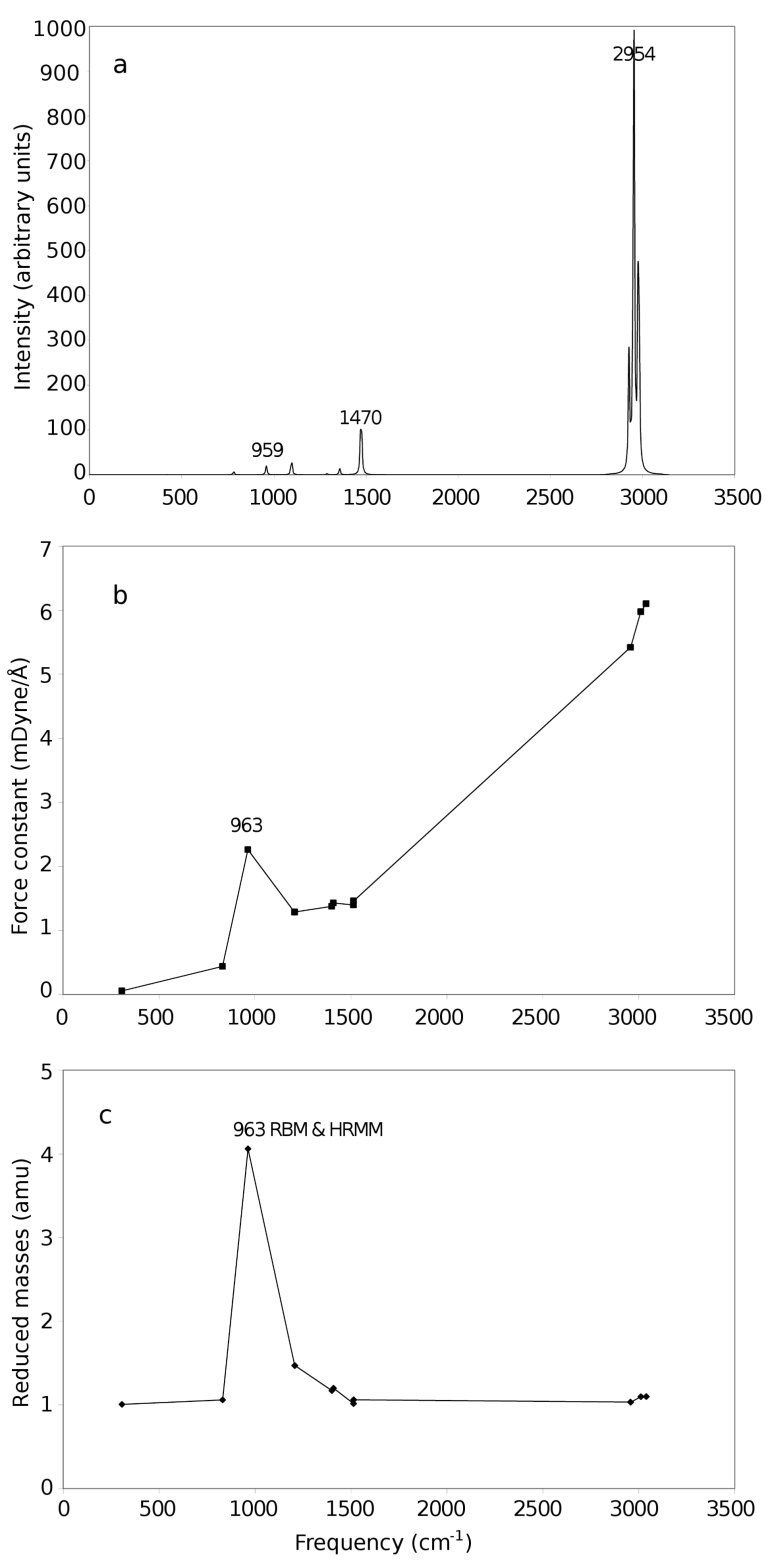
(a) Intensities, (b) force constants and (c) reduced masses of the ethane infrared spectrum as a function of frequency.

**Figure 4:**
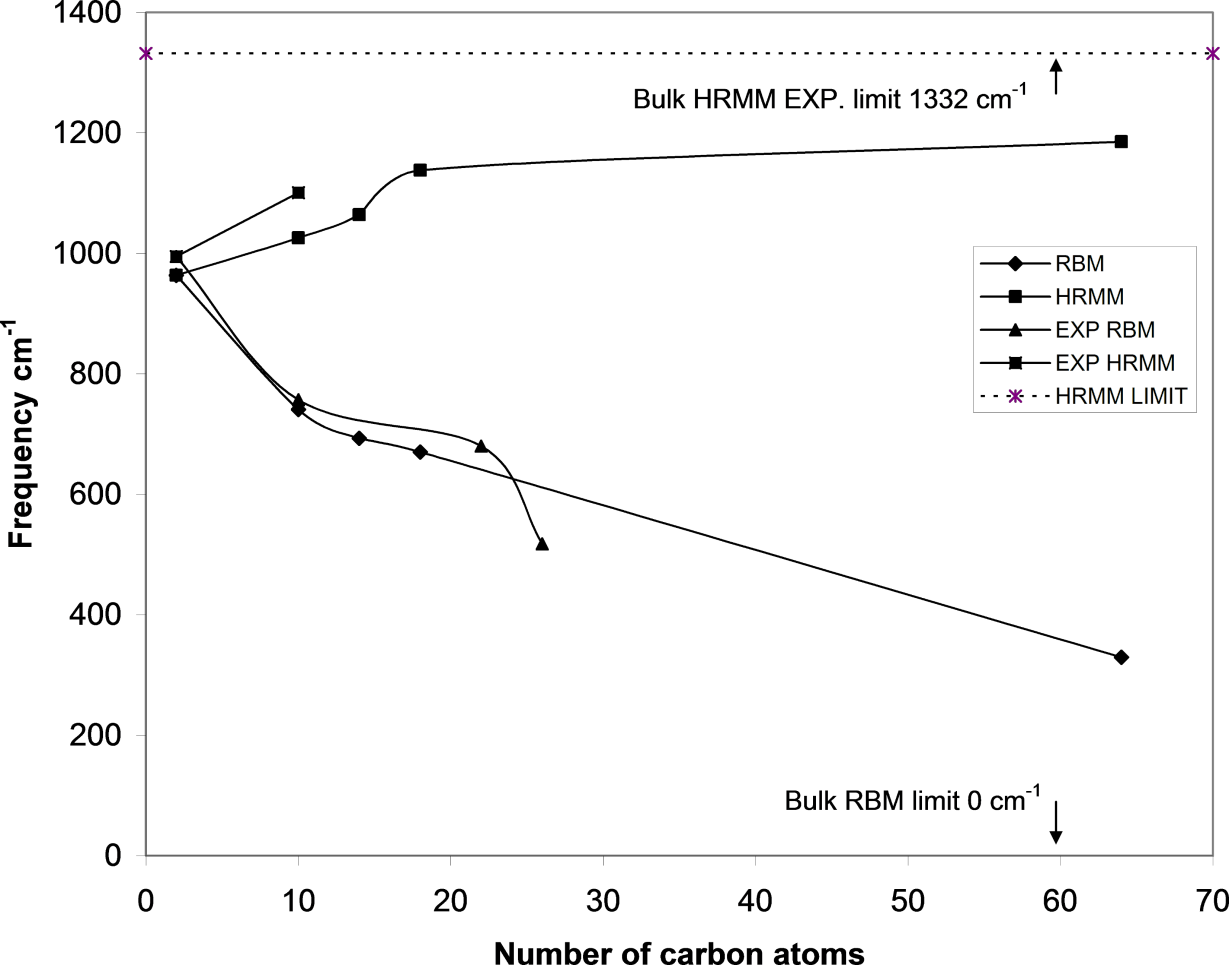
Frequencies of RBM and HRMM as a function of the number of carbon atoms. Experimental modes are from references [14,19]. The limits of these two modes in bulk diamond are shown.

**Figure 5:**
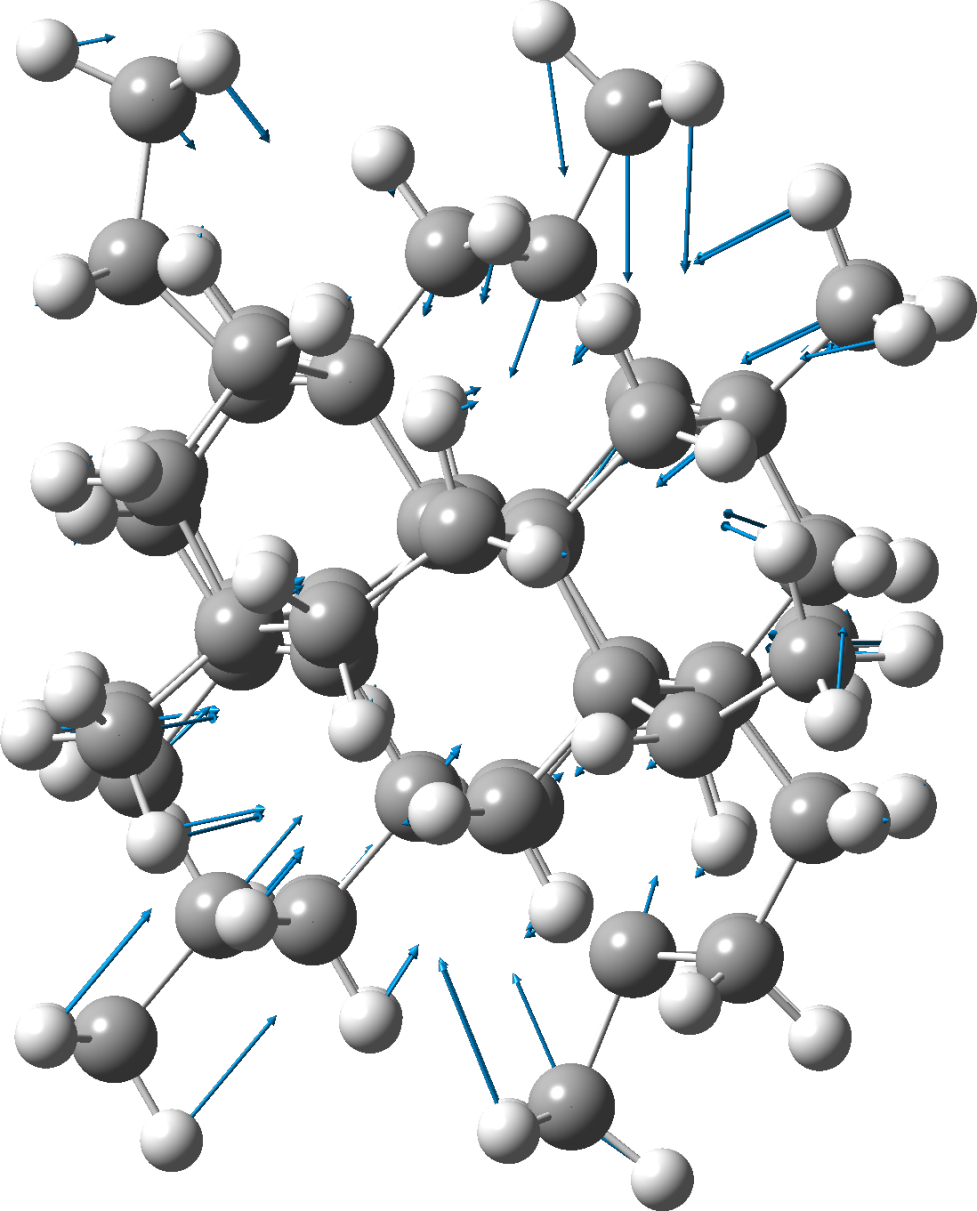
RBM displacement vectors in the diamond nanocrystal C_64_H_84_ at 330 cm^−1^.

Figure 2 shows the vibrational frequencies of adamantane. Figure 3 shows vibrational the frequencies of ethane. Figure 4 shows the variation of the highest reduced-mass mode (HRMM) of C–C vibrations and radial breathing mode (RBM) with the number of carbon atoms. Figure 5 shows the RBM displacement vectors in diamond nanocrystal C_64_H_84_ at 330 cm^−1^. For comparison of the present calculations a wide range of references exist for C–C and C–H vibrations [16–19]. Theoretical behavior of the radial breathing modes for nanomaterials can be found in reference [20].

## Discussion

In the first part of Figure 1 (0–1589 cm^−1^), we can note that most vibrations have a reduced mass of 2 atomic mass units (amu) or greater. This means a less active C–H vibrational contribution in this region. Most vibrations in the first region are C–C vibrations. The reduced mass formula of two particles of masses *m*_a_ and *m*_b_ is given by:

[1]
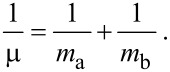


Although the above equation is for diatomic molecules, it can be used for the understanding of vibrational modes of other larger molecules. We can note from the above equation that the reduced mass of two carbon atoms (the mass of one carbon atom is approximately 12 amu) can be approximately 6 amu, which is the case of C–C vibrations and can be seen not to be exceeded in the reduced masses of Figure 1c. In the other two following figures (Figure 2c and Figure 3c) the highest reduced masses are 6.49 and 4.06 amu, respectively. This shows that the above rule is also approximately followed. The value of 6.49 amu of Figure 2c is due to movement of carbon atoms in phase with one of their bonded hydrogen atoms which makes their effective masses 13 instead of 12. On the other hand the C–H reduced vibrational mass is approximately 0.923 amu (the mass of one hydrogen atom is approximately 1 amu) and can be seen not to be violated in the three figures (Figures 1c–3c) by going to less than this value. All the reduced-mass points in the three Figures 1–3 are in between these two values (0.923 to 6.49 amu).

In order to determine peaks related to the bulk diamond structure and separate them from surface C–H vibrations, two conditions have to be met. The first condition is that the reduced mass has to be the closest to the reduced mass value of two atoms C–C at 6.5 amu (highest possible value in the spectrum), and the second condition is that it has to have a distinguishable high force constant that converges to the bulk diamond force constant at 4.7 mDyne/Å [16]. Two trends can be seen in the reduced masses of Figure 1c. The first trend is the acoustical branch that begins from the beginning of the spectrum and has peaks at 330, 516, 646, and 782 cm^−1^. This broad peak ends at nearly 819 cm^−1^ when the optical branch begins, which has strong oscillations between a lower limit above 1 amu reduced mass and highest values of reduced masses (HRMM) (5.25 amu) at 1185 cm^−1^. However, the highest intensity peaks in the above two regions have different positions than the highest reduced masses due to the overlap between neighboring peaks. We shall continue to refer to the value of the pure mode rather than the experimentally observed peak, which may move slightly due to overlapping. The range 300–700 cm^−1^ is the only wide range of frequencies that is totally free from the contaminating C–H vibrations, which have less than 2 amu of reduced mass. In our opinion this is the origin of the broad 500 cm^−1^ peak of diamond nanocrystals reported repeatedly in literature with varying explanations [2,4]. The existence of this broad peak is a definite signal of the existence of diamond nanocrystal structures.

From displacement vectors, the vibrational mode at 330 cm^−1^ peak is identified as the RBM in diamond nanocrystals (see Figure 5). This mode corresponds to radial expansion–contraction of the nanocrystal. From the value of the reduced mass and force constant, the HRMM peak at 1185 cm^−1^ is the distorted original experimental 1332 cm^−1^ diamond bulk line [2]. This phenomenon is termed as the vibrational red-shift effect in nanocrystals [21]. The idle strong bonds of bulk diamond are weakened in nanocrystals because of surface and reconstruction effects. Since surface effects penetrate at least three layers of the surface [1], the present nanocrystal, which has four layers between surface and core, will maintain a small number of idle tetrahedral bonds at the center or the core part of the present nanocrystal. The value of the force constant at this frequency (Figure 1b) supports this argument, having the exceptional value 4.4 mDyne/Å, close to the ideal bulk-diamond force constant mentioned earlier (4.7 mDyne/Å). As nanocrystals grow in size the intensity and force constant of this line increases and surface effects decrease, which enhances the strength of the bonds. Since the frequency of vibration is proportional to the force constant of the vibrating bond as given by the equation

[2]
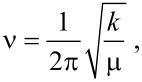


the frequency of the present 1185 cm^−1^ line will increase as the nanocrystals increase in size and head towards that of the bulk at 1332 cm^−1^. Note that many C–H vibrations interfere with the highest peak at 1185 cm^−1^ and continue to the end of the first part of Figure 1. This can be noted from the high oscillation in reduced masses at the end of first part of Figure 1c. The above range of frequencies are identified experimentally in nanodiamonds, such as the lines 1132, 1134, 1140, 1150, and 1240 cm^−1^ in references [2,4–5,7–8], respectively. These lines all belong partially to the originally distorted bulk diamond line at 1332 cm^−1^, which appears at different positions in different sizes of nanocrystals. C–H vibrations on the surface of diamond-like carbon or hydrocarbon molecules are near to the present C–H vibrations [16–18]. As an example, C–H vibrations that nearly match the low-frequency and reduced-mass vibrations in Figure 1 include those at 700, 755, 840, 910, 1030, 1075 and 1110 cm^−1^ as can be seen in Table 8 of [18]. These vibrations include different modes of vibrations such as rocking, bending, twisting and wagging [16–18].

The end of the first part of Figure 1 (1170 to 1589 cm^−1^) is distinguished from the beginning of Figure 1 by lower reduced masses and is mainly due to C–H vibrations that are coupled with C–C vibrations in the first part of the figure. C–H vibrations of similar frequencies on the surface of diamond-like carbon that match frequencies in Figure 1 include those at 1170, 1180, 1280, 1325, 1445, 1450 and 1490 cm^−1^ as can be seen in Table 8 of [18].

A frequency gap in the range 1589–2920 cm^−1^ is seen in Figure 1 compared to a frequency gap in the range 1490–2850 cm^−1^ in bulk diamond-like carbon or hydrocarbon molecules [18]. The differences can be attributed to various reasons, such as an internal structure effect, scale factor effect, size effects, etc.

An important feature of the second part of Figure 1 (range 2920–3174 cm^−1^) is that the reduced masses are all practically equal to 1. This is a sign that all modes are C–H modes. This includes symmetric and asymmetric stretching of CH_2_ and CH_3_ surface clusters, and symmetric deformation of the CH_3_ cluster. At the end of Figure 1 an sp^2^ hybridization mode is expected [18]. As in the case of the first part of Figure 1, many of the present frequencies are very near to their analogous lines in diamond-like carbon surfaces and hydrocarbon molecular frequencies such as the lines 2850, 2875, 2920cm^−1^, etc. [18]. Some of these lines are identified experimentally in diamond nanocrystals themselves, such as the 2857, 2930, 2971 cm^−1^, etc., lines [7].

In order to examine the variation of RBM and HRMM vibrational properties with the size transition from molecules to nanocrystals we investigated adamantane (C_10_H_16_), the smallest diamondoid, and ethane (C_2_H_6_), the smallest hydrocarbon, with C–C bonding using the same level of theory. As we can see from Figure 2 and Figure 3, the general shape of the intensity, force constant and reduced mass data are nearly the same as in Figure 1 with the exception of having a lower number of points and different peak heights. The breathing modes of adamantane and ethane are indicated in Figure 2 and Figure 3, respectively. The highest reduced-mass C–C lines are also indicated on these figures.

Figure 4 shows the frequency variation of RBM and HRMM as a function of the number of carbon atoms. In this figure we also included our theoretical results for RBM and HRMM of diamantane (C_14_H_20_) and triamantane (C_18_H_24_) using the same level of theory. The experimental values of the breathing and highest reduced-mass C–C modes of ethane and diamondoids [14,19], and the limits of these modes as nanocrystals grow in size are shown [2,20]. Since we have one C–C bond in ethane, the breathing mode in ethane is actually a one-dimensional stretching mode. The breathing-mode frequency is inversely proportional to the mean radius of the nanoparticles such that it approaches 0.0 cm^−1^ as these particles grow in size [20]. Experimental results of the HRMM C–C mode are limited. This can be attributed to the weak and gradually increasing intensity of this mode with increasing size. As an example, the theoretical intensities of the three present working examples C_2_H_6_, C_10_H_16_ and C_64_H_84_ are 0.0, 0.0001 and 1.1148 km/mole, respectively, as calculated by the present theory. This mode becomes the dominant mode in intensity in bulk diamond crystals at 1332 cm^−1^. Force constants for this mode also increase, as can be seen in the above figures, from 2.26 to 4.42 mDyne/Å as we go from ethane to C_64_H_84_. To the best of our knowledge, the trend of frequencies of these modes (RBM and HRMM) in Figure 4 as hydrocarbon molecules or diamond nanocrystals grow in size has not been presented previously in the literature. The two modes converge to one mode as the number of carbon atoms decreases to 2 atoms in ethane. Since RBM is actually an acoustical mode while the HRMM mode is an optical mode, Figure 4 also shows the collapse of acoustical and optical modes of bulk diamond to one mode at the ultimate molecular scale. To the best of our knowledge this “branch collapse” had not been reported before. In view of the recent applications of diamond nanocrystals that include medical [22] and industrial applications [23] involving the use of infrared spectroscopy, the present research has an extraordinary importance. The present method that incorporates the reduced-mass and force-constant analysis is sometimes used in solid-state physics and nanocrystals [21,24]. It is also used in large molecules, such as RNA [25–26].

## Conclusion

As concluding remarks, we can note that the present theory can adequately reproduce many of the experimental data of infrared vibrational frequencies. This includes the 330–1185 cm^−1^ modes in the C–C vibrational region. The region around the broad peak at 500 cm^−1^ has pure C–C vibrations and is a sign of diamond structure in nanocrystals. The present theory reproduces adequately various C–H vibrations, which include symmetric, asymmetric, wagging, scissor, rocking and twisting modes. It also reproduces the movement of the radial breathing mode and highest reduced-mass C–C mode as nanocrystals grow in size. The variation of the highest reduced-mass mode C–C vibrations from that of ethane at 963 cm^−1^ to that of bulk diamond at 1332 cm^−1^ is shown. The variation of the radial breathing mode from that of ethane at 963 cm^−1^ to that of bulk diamond at 0 cm^−1^ is also shown and is also found to coincide with experimental values. Acoustical and optical vibrational branches of bulk diamond are proved in the present work to approach each other at the nanoscale and collapse at the molecular limit.
